# Extracellular vesicles: mediators and biomarkers of pathology along CNS barriers

**DOI:** 10.1186/s12987-018-0104-7

**Published:** 2018-07-01

**Authors:** Servio H. Ramirez, Allison M. Andrews, Debayon Paul, Joel S. Pachter

**Affiliations:** 1 Department of Pathology and Laboratory Medicine, The Lewis Katz School of Medicine at Temple University, 3500 N Broad St, Philadelphia, PA 19140 USA; 2 Shriners Hospital Pediatric Research Center, Philadelphia, PA 19140 USA; 3 Center for Substance Abuse Research, The Lewis Katz School of Medicine at Temple University, Philadelphia, PA 19140 USA; 4 Department of Immunology, Blood-Brain Barrier Laboratory & Laser Capture Microdissection Core, UConn Health, 263 Farmington Ave., Farmington, CT 06070 USA

**Keywords:** Brain endothelial cells, Blood brain barrier, Extracellular vesicles, Microvesicles, Exosomes, Neuroinflammation

## Abstract

Extracellular vesicles (EVs) are heterogeneous, nano-sized vesicles that are shed into the blood and other body fluids, which disperse a variety of bioactive molecules (e.g., protein, mRNA, miRNA, DNA and lipids) to cellular targets over long and short distances. EVs are thought to be produced by nearly every cell type, however this review will focus specifically on EVs that originate from cells at the interface of CNS barriers. Highlighted topics include, EV biogenesis, the production of EVs in response to neuroinflammation, role in intercellular communication and their utility as a therapeutic platform. In this review, novel concepts regarding the use of EVs as biomarkers for BBB status and as facilitators for immune neuroinvasion are also discussed. Future directions and prospective are covered along with important unanswered questions in the field of CNS endothelial EV biology.

## Background

In the field of observation, chance favors only the prepared mind

*Louis Pasteur*

The observation by Chargaff and West in 1946, that a high-speed pellet derived from centrifuging blood contained factors that accelerated clotting of plasma—factors later recognized, in 1967, as bioactive vesicles and accorded the term “platelet dust” by Wolf [1]—has spawned a vast new field of biology with increasing ramifications in medicine and therapeutics. This advance would never have happened had early investigators not previously recognized the potential of “conditioned media” from cultured cells to impact growth and metabolism. It became clear that cell-derived factors released into extracellular fluid—both plasma and culture media—had nutritional value and powerful signaling potential. While many of these factors are simple organic solutes singularly secreted or transported, those of the platelet dust variety are complex, phospho-lipid, bilayer, plasma membranes that enclose cytoplasmic material and are shed from cells. Broad in derivation, composition, and activity, these membrane derivatives are now conventionally known as *extracellular vesicles*—more commonly referred to simply as EVs [2, 3].

As general reviews on EVs are vast, it is not the objective of this article to reiterate the already extensive literature on the well-recognized aspects of EV biogenesis and properties. Instead, given their burgeoning roles in neuroscience and neurology—including developmental biology [4, 5], neuroinflammation [6, 7], neuroinfection [8, 9], neurodegeneration [10, 11], brain tumors [12, 13], and psychiatric disease [14, 15]—the focus here is specifically on EVs that originate from and/or impact (CNS) barriers, and the prospects of EVs for diagnosis and treatment of neuroinflammatory/neurodegenerative diseases having CNS barrier involvement. However, where helpful in drawing certain analogies, references to EV activities along the peripheral vascular endothelium are included. And some general background in EVs is provided to aid in appreciating the roles of EVs in CNS barrier physiology, pathophysiology and therapy.

**Fig. 1:**
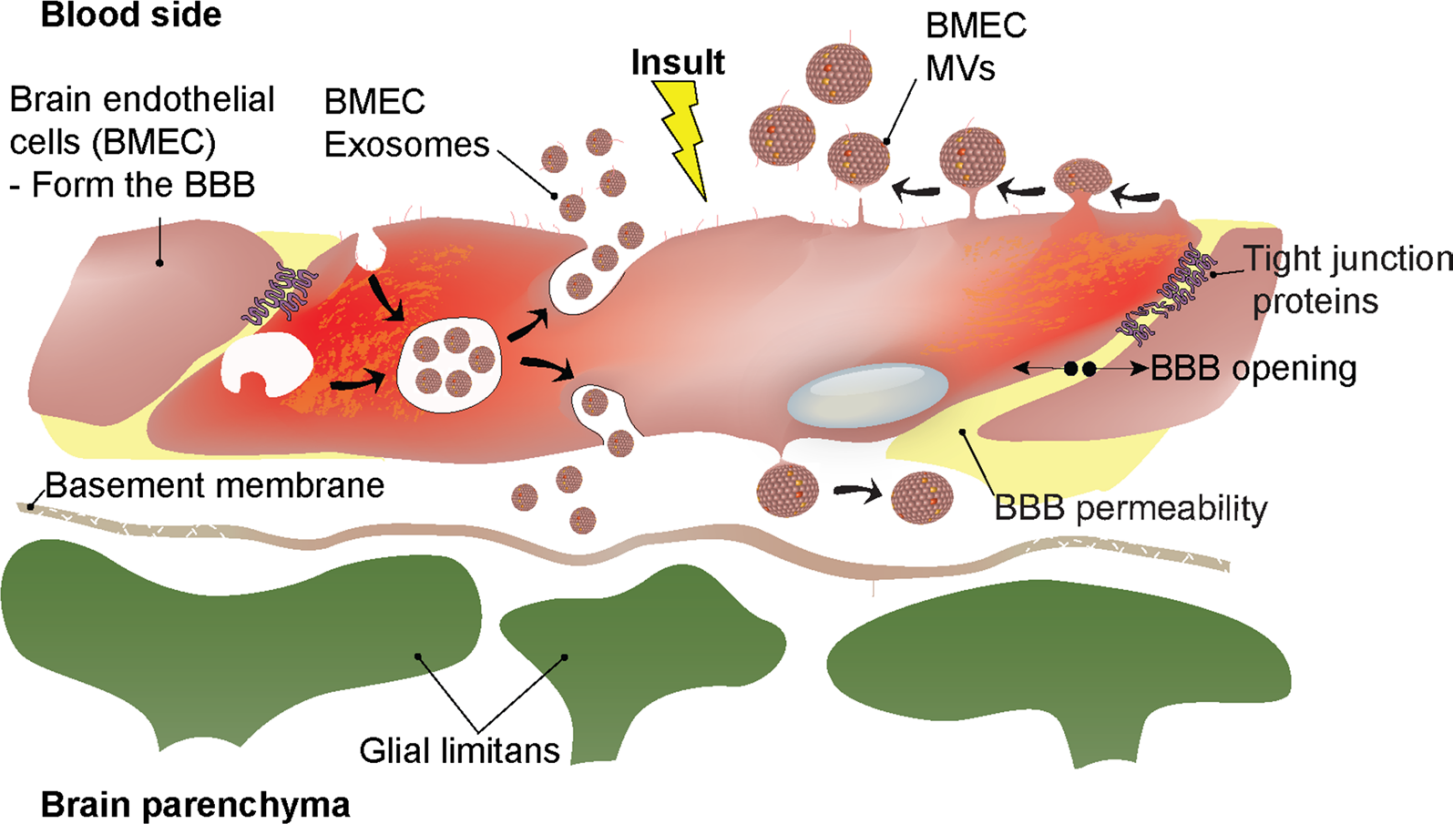
Microvesicle (MV) and exosome biogenesis in brain endothelial cells. Upon inflammatory stimuli, brain endothelial cells respond by releasing MVs (microvesicles) and exosomes into the bloodstream and/or in theory perivascularly. For exosomes, stimuli lead to internalization and formation of early endosomes that invaginate to create multivesicular bodies (MVB). For MVs, the vesicle is formed from budding of the plasma membrane.Vesicles are then released either into the blood or the brain parenchyma (theorized)

EVs are heterogeneous, nano-sized vesicles shed into blood and other body fluids by many cell types, which disperse a variety of bioactive molecules (e.g., protein, mRNA, miRNA, DNA and lipids) to cellular targets over long and short distances [16–19]. There are three EV subtypes conventionally recognized that are distinguished based on their respective size and route of biogenesis [3, 20, 21]. Two of these are released by live cells both constitutively and in response to stimulation, and are classified as *exosomes* (30–100 nm diameter) and *microvesicles* (100–1000 nm diameter (sometimes referred to as ‘microparticles’ [MPs]), though sometimes their respective sizes overlap (Fig. 1 and Table 1). Exosomes derive from in-budding of endosomes to form multi-vesicular bodies that fuse with the plasma membrane to release the membrane vesicles into the extracellular space. Microvesicles form by outward budding of the plasma membrane. A third subtype, *apoptotic bodies* (> 1000 nm), are released from dying cells and will not be a subject of this review. Besides originating via distinct processes, the varied subtype EVs—even from the same cell—carry different cargo within their membrane and luminal compartments and, a priori, execute different functions [22]. Recent evidence further suggests protein content of EVs might reflect the phenotype of the tissue of origin, such as the inflammatory state of the brain microvascular endothelium [23]. While all EVs tend to be highly enriched in tetraspanins, e.g., CD9, CD63, CD81, CD82 and CD151 [24], a consensus protein signature that faithfully distinguishes exosomes from microvesicles has not yet been realized. However, differential expression of proteins PDCC6IP and SDCB1 by exosomes, and ATP5A1, RACGAP1, and SEPT2 by microvesicles was observed in EVs released by cultured brain microvascular endothelial cells (BMECs)—which form the BBB—stimulated by the pro-inflammatory cytokine TNF-α [23] (Note: henceforth in this manuscript, in examples where brain endothelial cells are known to be specifically of microvessel origin, they will be referred to as BMEC; in other cases they will simply be noted as brain ECs). Exosomes from a human colon cancer cell line have further been shown to contain presumed “exosome marker proteins” Alix, TSG101, CD81 and CD63 not found in microvesicles isolated from culture supernatant of the same cells, while microvesicles showed selective enrichment of another 350 proteins [25]. And, there has also been report of unique miRNA sequences expressed by separate exosome and microvesicle populations isolated from blood of patients with clinically isolated syndrome (CSI), the first clinical evidence of CNS demyelination [26]. With refinements in isolation and characterization of EVs, there is expected to be growing awareness of additional unique markers for, and properties of, the different EV subtypes. These distinctions are likely to hold significance for physiological and pathophysiological roles of EVs at CNS barriers, and enable EVs to be exploited therapeutically and also serve as biomarkers of disease.

**Table 1:**
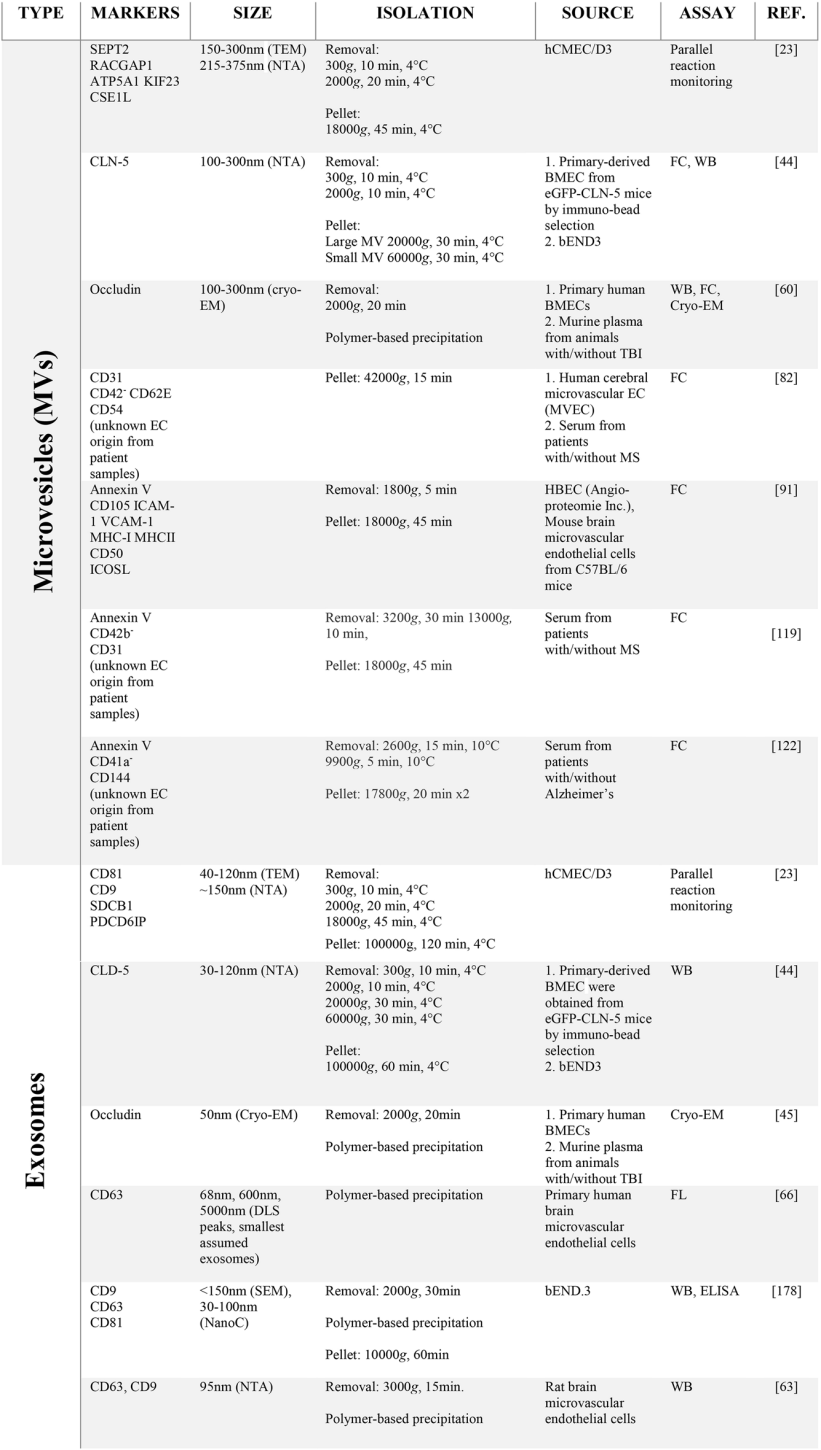

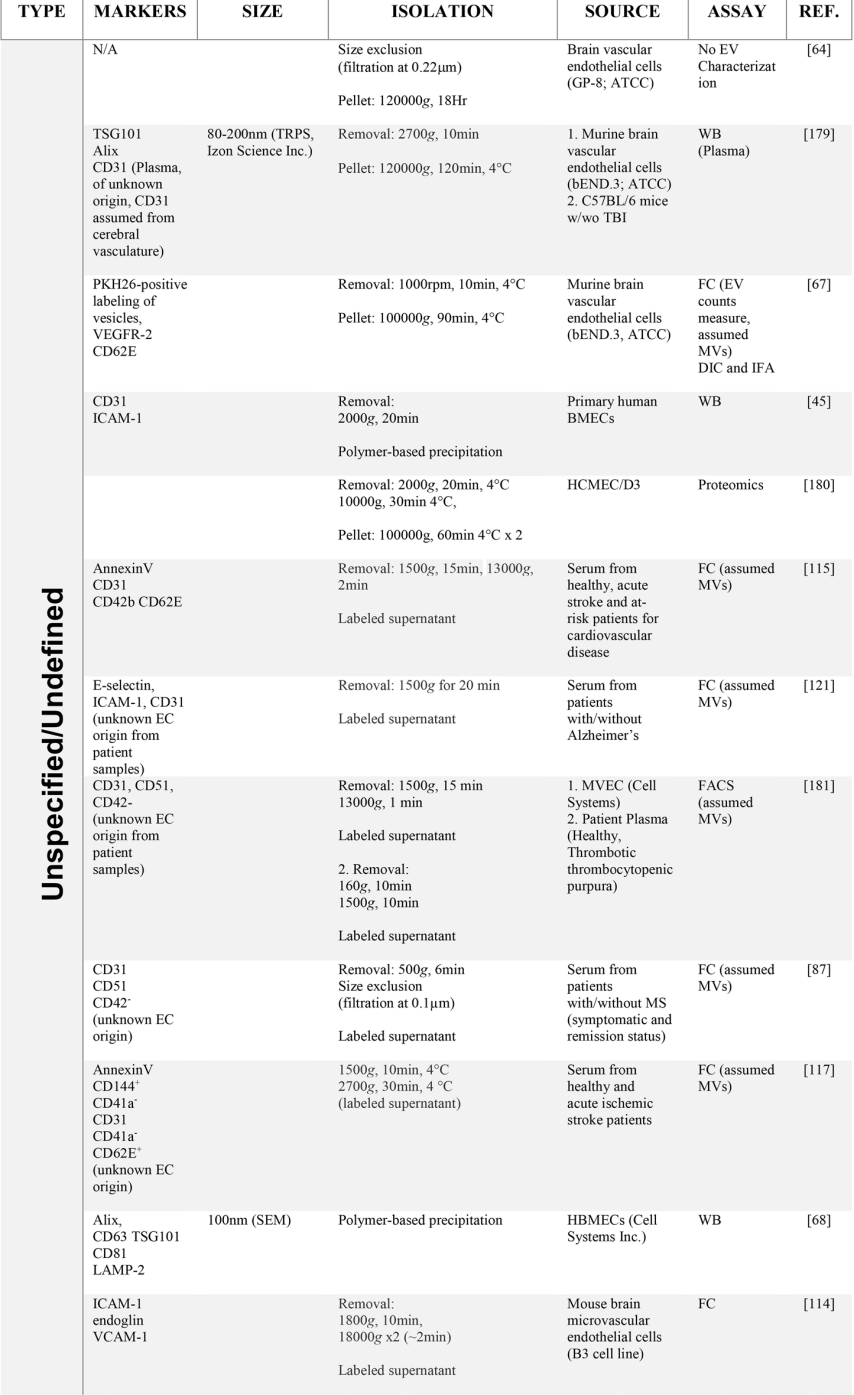
Markers, means of preparation, source (circulation or tissue culture), and assay of brain barrier-derived EVs according to subtype (exosomes or microvesicles)

EV subtype is designated based on crude sedimentation properties (EVs sedimenting at < 100,000×*g* are classified as microvesicles, while those sedimenting at > 100,000×*g* are classified as exosomes) or polymer-based precipitation (exosomes)

*TEM* transmission electron microscopy, *NTA* nanoparticle tracking analysis, *Cryo-EM* electron cryomicroscopy, *SEM* scanning electron microscopy, *DLS* dynamic light scattering, *DIC* differential interference contrast microscopy, *TRPS* tunable resistive pulse sensing, *FC* flow cytometry, *WB* western blot, *FL* fluorescence labeling, *MS* multiple sclerosis

There are several types of CNS barriers. Perhaps the most widely recognized is the blood–brain barrier (BBB), which lies at the level of parenchymal microvessels and is formed by a monolayer of specialized endothelial cells characterized by high-resistance tight junctions (TJs) and subtended by the *glia limitans*. For the purposes of this review, we will consider the blood–spinal cord barrier in the same context as the BBB, though subtle differences between the two have been noted [27]. The blood–cerebrospinal barrier (BCSFB) is comprised of epithelial cells of the choroid plexus (localized in the brain ventricles) that separate blood–borne elements from the cerebrospinal fluid (CSF) [28]. A third type CNS barrier, the blood–leptomeningeal barrier (BLMB), is formed by a monolayer of endothelial cells of the microvessels coursing through the pia mater and overlying subarachnoid space (SAS), and may be considered another type of BCSFB [29]. The epithelioid pia matter, itself, has been suggested to act as yet an additional CNS barrier, regulating solute and cell traffic between the CSF in the SAS and the sub-pial parenchyma [30, 31]. While technically outside the CNS, there also exists a blood–retinal barrier (BRB), which consists of retinal pigment epithelial cells and retinal capillary endothelial cells each tightly connected by specialized junctional complexes similar to those found at the BBB [32]. This review will highlight known and prospective interactions of EVs with these different CNS barriers. The initial focus will be on the BBB and EVs derived from the microvascular endothelium comprising this barrier.

## EVs in endothelial biology: inside and outside the CNS

What might be considered the earliest references to EVs from endothelial cells were described by Hamilton et al. [33] and Patel et al. [34]. These pioneering studies were then followed by a hiatus in endothelial EV research for nearly a decade. The revival of endothelial-EV research lead by Combes et al. [35] proceeded with a quickening pace and with focus on the potential for EVs as markers of cardiovascular damage. These studies mainly focused on the larger microvesicles, with many in the field commonly referring to them as “EMPs” or endothelial microparticles [36]. The study of endothelial-derived exosomes, however, has lagged behind until more recently.

Given the extensiveness of the vasculature, similarities and differences exist amongst its derivative branches. Nearly all endothelial cells are thought to express common “endothelial markers” such as PECAM-1 (CD31), von Willibrand factor (vWF), CD34, and CD144 [37–39]. The field of cardiovascular research has exploited this to define cardiovascular dysfunction and disease by the characterization of endothelial-derived EVs separately from other cell-derived EVs (i.e., immune cells, platelets). Due to the overlap in some protein expression, it is common to use a combination of markers, which includes multiple surface proteins such as adhesion molecules (PECAM-1, ICAM-1, E-selectin, VCAM-1, endoglin, MCAM), cadherins (i.e., VE-cadherin), and integrins [40–42]. Additionally, Annexin-V is also widely used because of known mechanisms of microvesicle release involving phosphatidylserine [43]. While analysis of endothelial EVs based on these common endothelial markers can give a picture of overall vascular health, it may also be possible to identify the health of a particular vascular network—for example, the cerebrovasculature. Combining general endothelial markers with specific vascular domain markers could potentially fine-tune our use of EVs. In the case of BMEC, there are a number of proteins and transporters that are highly enriched or brain specific [44, 45], and might be exploited to confirm a CNS microvascular origin for circulating EVs. For example, a recent study by Andrews et al. demonstrated the increased presence of the TJ protein, occludin, on EVs following TBI [45]. In another study circulating ECs were shown to carry a different TJ protein, claudin-5, in an animal model of MS [44]. Indeed, these studies show that detection of endothelial markers and of proteins highly expressed in the cerebrovasculature, could provide a means to interrogate the health of CNS endothelial barriers, such as those that form the BBB. However, as both occludin and CLN-5 are also expressed by peripheral endothelial cells, expression of these proteins alone is insufficient to confirm the CNS derivation of EVs. Co-expression of transporter proteins critical to BBB function might offer the best an option for specifically identifying BMEC-derived EVs. Interestingly, endothelial markers and barrier endothelium enriched proteins can be found on both forms of EVs. Given how much EVs could reveal about vascular health, future investigation in which EC-derived microvesicles versus exosomes are compared for differential release as a function of time could provide a snapshot of the respective relevance of each EV subtype in physiology and pathophysiology. It is likely that the kinetics of EC release may differ with other cell types due to their interface with the bloodstream, and their larger and flatter morphology (i.e., surface area) that could provide a higher membrane reserve than other cells.

## Biogenesis and molecular composition of exosomes and microvesicles

### Exosomes

As stated earlier, circulating endothelial EVs are elevated in conditions of vascular dysfunction, and their presence can be used as biosignatures of vascular pathology [46]. The change in endothelial status leading to the release of EVs may be reflective of maladaptive vascular tone, immune recruitment, and/or thrombosis [47]. Specifically, triggers that mediate EV release may involve either inflammatory cytokines, ROS, lipopolysaccharides, thrombin, hypoxia or aberrant shear stress or a combination thereof. Cellular insults subsequently can induce the release of exosomes and/or microvesicles. Because exosomes arise from within MVB, which in turn originate from the maturation process of intraluminal vesicles contained within early to late endosomes [48], exosomes will bear proteins characteristic of late endosomes such as CD63, LAMP1 and LAMP2. Importantly, the early endosomal proteins, Rab4 or Rab5, may also be found in exosomes, suggesting various routing mechanisms affecting vesicle exocytosis [49]. A key aspect in the formation of MVB is the involvement of the endosomal sorting complex required for transport (ESCRT) proteins (reviewed in [50]). These family of proteins form intracellular complexes comprised of: ESCRT-0, -I, -II, -III and the Vps4 complex. Cargo composition within exosomes is highly context dependent which is affected by the cell type, status of cell cycle, and exposure to environmental cues. However, in general the heterogeneity of the cargo is a mixture of genetic information (such as non-coding RNAs), proteins and lipids [50]. It is important to mention that although much is known about the molecular machinery involved in exosome biogenesis, much remains to be done to validate if the same processes apply to brain EC exosome production.

### Microvesicles

Certain members of the endocytic pathway are recoverable in 10,000 x g pellets of cell-conditioned media from neuroblastoma cells, suggesting they are likewise involved in microvesicle formation, for example the ESCRT family of proteins Alix and TSG101. Recently, the ADP-ribosylation factor 6 (ARF6) has also been identified as important in mediating cytoskeletal changes that allows for microvesicle biogenesis. SCRT complexes are formed at the plasma membrane at areas of disrupted plasma membrane, which leads to the exposure of phosphatidylserine on the outer leaflet [51]. Recruitment and activation of flippase, floppase and scramblase mediate the final steps of the budding process [52]. The vertical packaging of cargo by microvesicles can include a milieu of bioactive agents that includes proteins, metabolites, DNA, mRNA, and miRNA (reviewed in [53]). The above processes of EV release appear to be universally involved in all cell types. However, similar to the need for comparative studies of exosome biogenesis between other cell types and brain ECs, the same can be said about microvesicle formation between brain ECs and other cell types.

## EC-derived EVs: role in CNS pathology

### Cell–cell communication (from brain ECs toward the brain parenchyma)

One of the main proposed functions of EVs is an involvement in cell-to-cell communication and signaling. Numerous reports have examined this intercellular signaling role in terms of both positive and negative effector function [54], although these studies concerned pathways operating outside the brain. Communication between the blood and the brain is critical due to the high metabolic demand of neurons and the need for the BBB to control trafficking of molecules and ions [55]. Consequently, EVs could potentially serve as a major mediator in these communications between the cells of the neurovascular unit (NVU).

As all cells of the NVU have the potential to communicate unidirectionally or bidirectionally among several cell types, this raises the question whether EVs have an exclusive target or engage in more promiscuous communications. A few studies have examined this in vitro and, from the scant results so far, we are beginning see a picture of cell specific cross-talk. Exosomes secreted from stimulated cortical neurons were shown to bind to and be endocytosed by only neurons. In contrast, neuroblastoma exosomes, which bind to neurons and glial cells indiscriminately, were only endocytosed by glial cells [56]. Furthermore, EVs released from metabolically labeled astrocytes/neurons were found in unlabeled endothelial cells, though this preferentially occurred with astrocyte-derived EVs [55].

The endothelium has long been known to secrete factors that act in a paracrine (acting on neighbors) as well as autocrine (acting on self) fashion. A priori, the use of EVs for endothelial communication seems logical. In paracrine, two forms of intercellular communications may be considered, direct and indirect. Direct communication occurs when ligands on the surface of EVs directly interact with receptors on the target cell. Whereas, indirect communication may be considered as a result of cargo proteins binding with cellular components of the target cell after EV internalization and decapsulation [57]. Moreover, since BMECs are also polarized cells, intercellular communication must also be considered within this context. Thus, the distinct luminal and abluminal protein expression, likely affecting the protein contents of EVs and their role in cell signaling. It also stands to reason, that cell polarization could affect protein cargo sorting and distribution—a key parameter to consider when performing studies in vitro [58]. A similar possibility could also result when using brain ECs under conditions of fluid flow and shear stress, since polarization is known to occur via mechanotransduction and could affect protein and gene expression during EV production [59]. Presently, no published studies have yet examined the impact of fluid dynamics on the release of EVs from the abluminal vs luminal endothelial surface of BMEC, and the cargo within these respective EV populations.

EVs released by ECs into the bloodstream have several potential targets including neighboring ECs, immune cells and downstream organ systems. Endothelial-derived EVs have been shown to affect the function of ECs from the same vascular bed [60–62]. Although these studies involve peripheral ECs, it would not be unexpected for brain ECs to also exhibit signaling changes in response to EC-derived vesicles. In fact, Kurachi et al. showed that EVs derived from various ECs (aortic, brain, umbilical) have similar effects on oligodendrocytes [63], which suggests potential similarities among EVs from different vascular beds in executing some actions. Although cross-talk between the vascular lineages has not been examined, it has been demonstrated by adoptive transfer of BMEC-derived EVs (in vivo) that these vesicles can have an effect on other organ systems such as the liver [64, 65].

Studying the fate and effect of endothelial EVs in the brain parenchyma remains more challenging than those appearing in the blood. However, several in vitro studies have begun examining the effects of brain EC-derived EVs on neighboring cells such as astrocytes, pericytes and oligodendrocytes. Due to their anatomical proximity within the NVU, astrocytes and pericytes are the most likely direct target of EVs released by BMECs. However, further investigations into the transport of vesicles in the extracellular space could reveal more direct contact with ECs without an intermediary cell. Recently, András et al. showed that BMEC-derived ECs treated with Aβ, triggered re-packaging of the Aβ in vesicles of various sizes [66]. This process was elevated in the context of exposure of cells to HIV particles, which induced the release of more EVs. Furthermore, these EVs were easily taken up by both astrocytes and pericytes—though analysis of resulting effects in the recipient cells was not pursued. The same group performed adoptive transfer experiments to investigate how the BBB may mediate Aβ trafficking into the brain in vivo. BMEC-derived EVs loaded with Aβ were seen to localize with the BBB and in the brain parenchyma [66]. Uptake of BMEC-derived-EVs by pericytes was also shown by Yamamoto et al. The results revealed that such EVs were produced in response to an LPS challenge and induced pericyte upregulation of VEGFB mRNA and protein expression [67]. The upregulation of VEGFB was suggested to mediate pathological angiogenesis and/or vascular leakage. Both of these studies examined vesicles from brain ECs under pro-inflammatory insults, which collectively reflect most studies on general EC vesicles produced under inflammatory conditions. In contrast, a few reports have shown positive effects of BMEC-derived EVs when produced under basal or non-inflammatory conditions [63, 68]. In the case of Kurachi et al., the authors examined the effect of basally produced BMEC-derived EVs that were then taken up by oligodendrocytes and inhibited apoptosis. Interestingly, this effect was not limited to BMEC-derived ECs, as EVs from other ECs (aortic, umbilical) also had a similar effect. Positive effects have also been seen from BMEC-derived EVs produced in response to TLR3 signaling. Li et al. showed these EVs contained antiviral factors that, when internalized by HIV infected macrophages, resulted in suppression of HIV reverse transcriptase activity [68]. Future studies will no doubt expand on our currently limited understanding of the positive and negative effects of EVs in the CNS.

### EVs as facilitators of immune cell entry into the CNS

#### Endothelial EVs are associated with inflammatory events along the BBB

Multiple sclerosis (MS) is a *multi*-*component*, *neurodegenerative*, *demyelinating* disease of the CNS, characterized histopathologically by focal inflammatory infiltrates, demyelinating plaques and axonal damage [69–71]. In the most common form of the disease, relapsing/remitting MS (RRMS), patients experience discrete neurological attacks (relapses) separated by a complete or partial return to periods of normal function (remissions). Another manifestation of MS is disruption of the BBB, which is viewed both as a possible secondary effect of neuroinflammation and leukocyte transmigration, and as integral to pathogenesis [72–74]. In fact, BBB disruption is considered an early feature of the disease [75]—possibly even preceding immune cell infiltration [76]. Several observations collectively allude to the possibility EVs play a role in regulating leukocyte CNS infiltration during MS [7, 77, 78]. Key, is the increasing evidence of interactions among EVs, leukocytes, and endothelial cells in neuroinflammatory conditions. The earliest indication of this relationship is the altered appearance of endothelial EVs in the blood of MS patients. Notably, inflammation of the endothelium, which is indicated in MS brain microvasculature by up-regulation of the adhesion molecule VCAM-1 [79] and aberrant distribution of TJ proteins occludin, ZO-1 and JAM-A [80, 81] in active lesions is a potent trigger for endothelial cells, in general, to release EVs [41, 43, 82–84]. A relationship between adhesion of leukocytes to peripheral endothelial cells and release of EVs by the latter has also been described in atherosclerosis ([85]), a condition displaying evidence of peripheral vascular inflammation. Accordingly, release of EVs by endothelial cells is thought to reflect endothelial activation and/or stress [43, 86], and has been reported elevated in MS [87]—the EVs in question being designated EMPs and identified as two subtypes by their respective expression of CD31 (PECAM-1) and CD51 (vitronectin receptor). CD31 + EMPs were elevated only during exacerbation, where their presence correlated positively with gadolinium-enhanced lesions, and then returned to control value at remission, while CD52 + EMPs remained elevated during all phases of disease, prompting the authors to conclude the former EV subtype is a marker of acute endothelial injury, whereas the latter reflects chronic injury. Further highlighting the correlation with inflammatory state, plasma levels of CD31 + EMPs were significantly reduced in MS patients treated with IFN-β1a, a standard therapy for RRMS, and declined in the same direction as clinical disability [88]. Such inflammatory responses are not limited to MS, as intracerebral injection of IL1-β into rats was also found to prompt release of endothelial cell-derived EVs, which became sequestered in the liver and mediated the acute-phase response and sickness behavior associated with CNS inflammation [64]. While it is important to keep in mind that the circulating and liver-bound EVs described in these studies were not explicitly identified as coming from the CNS microvasculature, when EVs released from IL1-β-treated BMEC cultures where injected back into rats, they elicited the same behavioral pathology.

#### EVs bind to/stimulate leukocytes during neuroinflammation

**Fig. 2:**
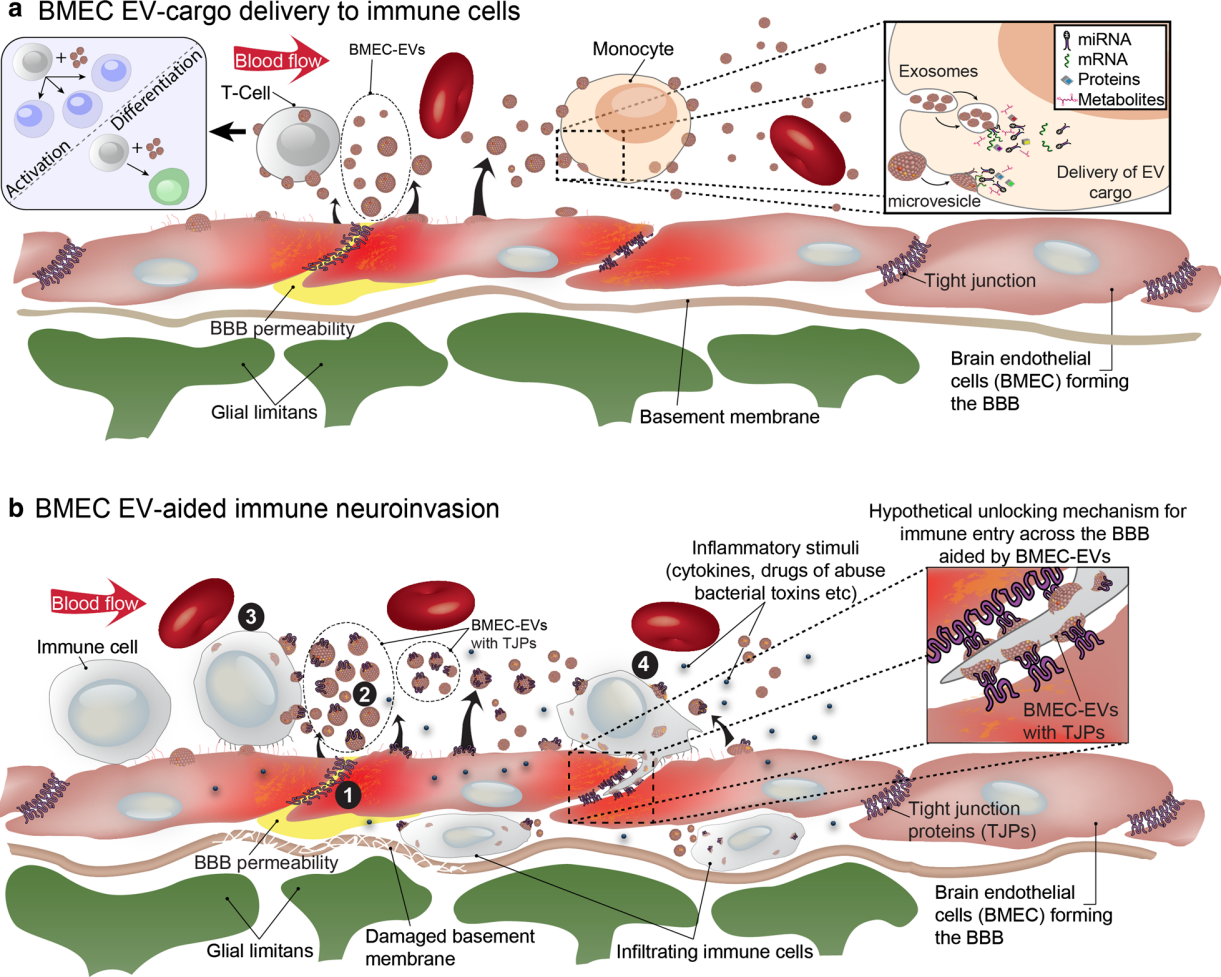
Hypothetical schematic model of EV-cargo delivery to immune cells and EV-aided immune transendothelial migration. **a** Exomes produced by the BMEC, can deliver its cargo via cellular endocytic internalization. Conversely, microvesicle fusion with cell membranes of the target cell facilities emptying of their cargo. The contents in EVs can be highly heterogenous (in terms of composition and amount) although it’s generally recognized that proteins, genetic material, and metabolites encompass most of its components. The biological consequences that BMEC-EVs could have on immune cells remains largely unknown. However, recent evidence suggests that EVs could affect T cell activation and differentiation status. **b** The presence of inflammatory mediators activates the brain endothelium and results in localized blood–brain barrier instability (BBB) (*1*). Subsequent hyperpermeability at the BBB may be explained by the temporal production of microvesicles and exosomes (EVs) (*2*). Importantly, brain endothelial derived EVs are known to contain tight junction proteins in their cargo [44, 45]. Although inflammatory mediators, drugs of abuse etc., induce the biogenesis of EVs from the endothelium, we propose that only activated immune cells can efficiently use these vesicles for gaining entry into the CNS. In the case illustrated, we hypothesize activated monocytes bind EVs containing TJ proteins (*3*), which then allows for direct “unzipping” of intercellular tight junction complexes at the BBB (*4*), leading to immune cell access to the perivascular space

Once discharged from endothelial cells, circulating EVs can bind to leukocytes and influence their activity [89], though, once again, the vascular bed of origin of these EVs is uncertain. The physical closeness between endothelial cells and marginating leukocytes could conceivably allow for EVs released by endothelial cells to more effectively interact with leukocytes in a juxtacrine (i.e., contact-dependent) manner, without being diluted in the circulation or affecting off-target sites (Fig. 2a). EV:leukocyte binding was initially revealed by detection of endothelial EV:monocyte complexes in blood of MS patients [82], and formation of similar complexes in vitro. That these complexes were significantly elevated during disease relapse versus remission or compared to controls, and correlated with gadolinium enhancement on MRI, further underscore their clinical relevance as harbingers of neuroinflammation and possibly a role for endothelial EVs as key players in the inflammatory process. In keeping with this inflammatory profile, EVs obtained from the supernatant cultures of endothelial cells stimulated with the pro-inflammatory cytokine TNF-α facilitated monocyte chemotaxis [90]. EVs released by TNF-α stimulation of cultured BMECs have also been shown to interact with and stimulate proliferation of T cells [91]. A pro-inflammatory impact on T cells outside the CNS has been observed as well, with endothelial EVs (identified as CD31^+^/CD42^−^, with diameters < 1 μm) in plasma from patients with acute coronary syndrome positively correlated with IFN-γ, possibly upregulating the differentiation and function of Th1 cells through increasing the expression of T-bet mRNA and protein [92]. In contrast, endothelial EVs can additionally suppress monocyte activation via transferring anti-inflammatory miRNAs, suggesting EVs can both promote and inhibit inflammation via differential actions on leukocytes [93].

Subpopulations of EVs released from BMECs in culture [92], and into the circulation by unidentified endothelial sources in vivo [94], have also been reported to contain various adhesion proteins. Moreover, such proteins appear more prominently expressed in exosomes versus microvesicles, in EVs released from TNF-α-stimulated BMEC [23]. While the physiological significance of these particular EVs remains to be clarified by experimentation, their detection suggests possible further EV interactions with leukocytes. For example, EVs expressing TJ protein CLN-5 [44] have been isolated from both BMEC cultures and peripheral blood of mice with experimental autoimmune encephalomyelitis (EAE), an animal model of demyelinating neuroinflammation that recapitulates seminal facets of MS—including leukocyte migration across the BBB. And another TJ protein, occludin [45], likewise has been detected on EVs released from cultured BMEC, and in the circulation following TBI, a condition, like MS/EAE, associated with neuroinflammation and CNS leukocyte infiltration. As peripheral blood leukocytes from MS patients were reported to increase ectopic expression of both CLN-5 and occludin during disease relapse, and down regulate them in remission or following anti-inflammatory therapy [95], it is tempting to speculate EVs mediate transfer of TJ proteins from endothelial cells—in particular BMEC—to leukocytes during neuroinflammation. This possibility is reinforced by findings that eGFP-labeled CLN-5 expressed exclusively by endothelial cells could be transferred to leukocytes during EAE, while eGFP-CLN-5^+^ EVs isolated from plasma of these mice also bound to leukocytes in vitro [44]. What purpose leukocyte-associated TJ proteins serve awaits resolution, but they have been argued to play an accessory role in leukocyte transendothelial migration (TEM) via a *zipper mechanism*, whereby inter-endothelial junctional contacts are temporarily replaced by homophilic interactions between leukocyte and endothelial junctional proteins [95, 96] (Fig. 2b). Because basal high-level expression of TJ proteins on circulating leukocytes would be a risk for cell aggregation in blood, focal binding of endothelial-derived, TJ protein^+^ EVs to leukocytes at the BBB during neuroinflammation could, conceivably, avert this problem.

#### EVs are associated with enhanced TEM

That binding of endothelial EVs to leukocytes might facilitate leukocyte extravasation is lent credence by the observation endothelial EV:monocyte complexes obtained from blood of MS patients showed enhanced ability, when compared to unconjugated leukocytes, in migrating across monolayer cultures of human BMEC [36, 77]. A close, physical association among endothelial cells, EVs and leukocytes during EAE—at sites of leukocyte margination along spinal venules—was additionally demonstrated using serial electron microscopy and 3D contour-based surface reconstruction [44] and provides more suggestive evidence supporting a role for endothelial-derived EVs in regulating leukocyte behavior during neuroinflammation. However, confirmation of a direct role for these EVs in promoting leukocyte TEM, awaits demonstration that their binding to, and not merely association with, a given leukocyte population stimulates the extravasation process.

#### EVs and neuropathogenesis of human immunodeficiency virus (HIV)

**Fig. 3:**
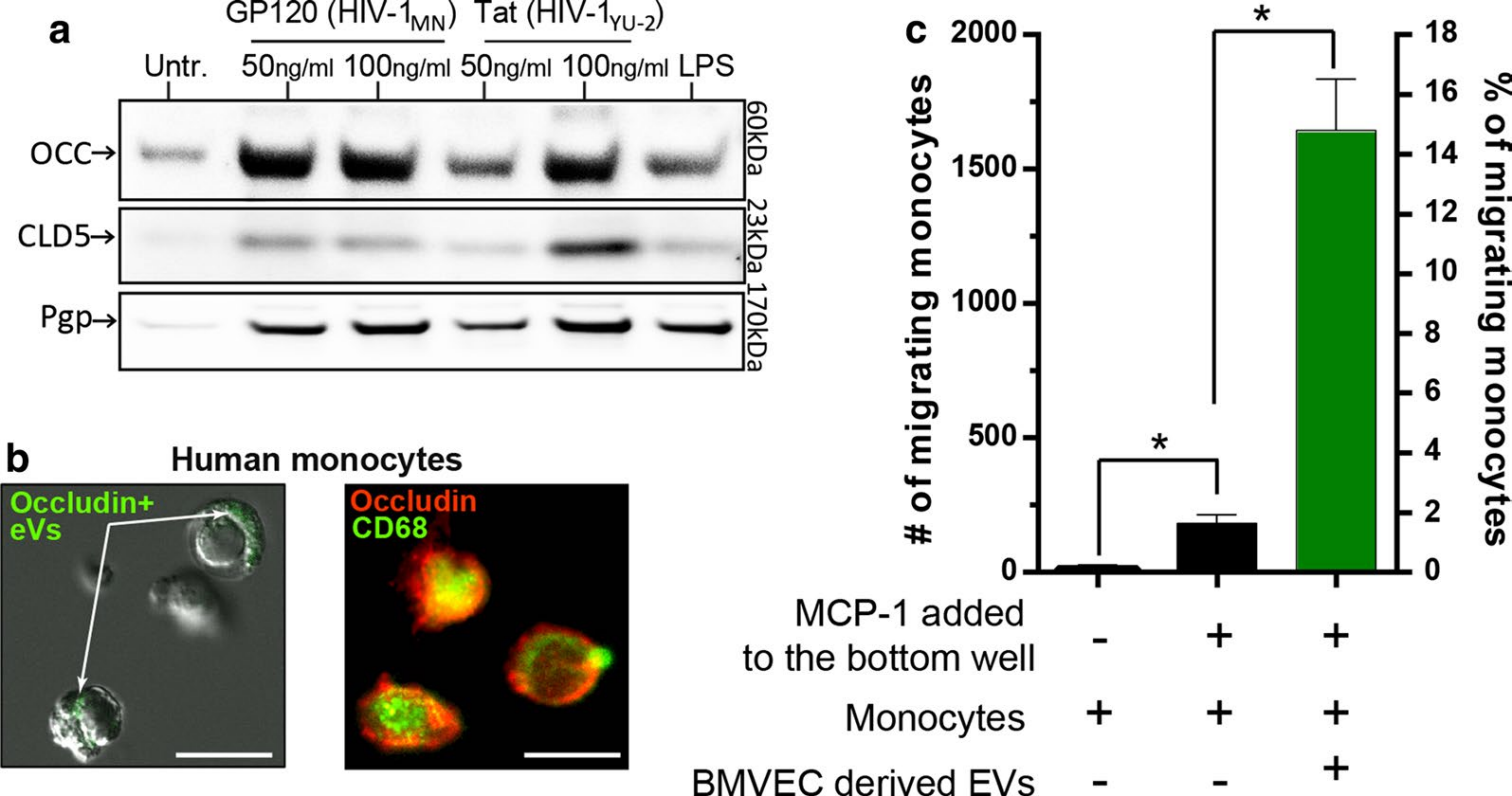
BMEC EVs and their effect on immune cell migration in the context of HIV. **a** Analysis of TJ proteins and endothelial markers in MVs released by BMECs following HIV virotoxin exposure. HIV virotoxins, gp120MN and TatYU2 induce increased levels of TJP release on MVs and of transporter protein p-glycoprotein, Pgp. **b** Representative image of immunofluorescence showing colocalization of TJP occludin with monocytic marker after incubation of monocytes with BMEC-EVs (right). To demonstrate exogenous occludin expression due to EV interaction/uptake, monocytes were incubated with EVs isolated from BMECs transfected with occludin-GFP (left). Scale bar = 10 microns. **c** Monocytes exhibit increased transendothelial migration when exposed to BMEC derived EVs. Migration assays were conducted for 4 h during which fluorescently labeled monocytes w/o EVs were introduced to BMECs grown on fluoroBlok inserts (Corning©). Monocytes were isolated from blood filters using a PAN-monocyte isolate kit (Miltenyi Biotech). EVs were isolated from BMECs treated for 24 h with 50 ng/ml of HIV-1 Tat (YU2). Isolated EVs were incubated with 1x10^5^ monocytes. MCP-1 at 20 ng/ml was included where indicated to initiate the migration. Results from counts of migrated monocytes are shown as the average + SEM, (*) P < 0.05

Despite treatment of HIV infection with antiretroviral therapy (ART), the CNS is still a major target and reservoir of the virus. Recent reports indicate that even in the presence of ART, chronic neuroinflammation cannot be fully prevented, leading to the development of HIV-1-associated neurocognitive disorders (HAND) in HIV-infected patients through increased neuroinflammation and BBB/vascular disruption [97–100]. Subsets of activated monocytes that express CD16 and/or CD163 are expanded both in HIV-infected individuals and in Rhesus macaques infected with simian immunodeficiency virus (SIV) [101], an HIV-like virus that can infect monkeys and apes and causes an AIDS-like disease. In fact, CD16 + monocytes serve as a continuous means of trafficking HIV from the periphery into the brain [102]. While these cells are refractory to replication, they differentiate into macrophages in the brain which can effectively sequester and replicate the virus. Although the production of EVs from various cell types are a characterized occurrence during HIV pathogenesis, not much is known about the EV response from brain endothelial cells during the course of viral infection. The only report that points to this possibility notes EVs are significantly released by cultured BMEC from the human-derived cell line, hCMEC/D3, when these cells are exposed to HIV viral particles. Although particle concentration was analyzed, the cargo from these cells was not. Figure 3 corroborates the above findings, showing generation of EVs following exposure of primary human BMECs to recombinant HIV proteins. Importantly, the EVs isolated showed the presence of p-glycoprotein (Pgp) and TJ proteins, occludin and claudin-5. These results suggest that BMECs are sensitive to HIV-1 viral proteins and respond by generating EVs that contain proteins associated with the tight junction complex and transport barrier. Moreover, using a migration assay with isolated human monocytes exposed to BMEC-derived EVs, the number of monocytes crossing BMEC monolayers significantly increased compared to monocytes not treated with EVs. These analyses are consistent with the report by Paul et al. [44], which showed endothelial-derived CLN-5 being transferred to leukocytes both in the blood and CNS of mice with EAE, as well as BMEC-derived CLN-5 + EVs able to bind to leukocytes in vitro. Thus, it is possible that EV-assisted immunoinvasion by HIV infected immune cells may represent a previously unrecognized cellular mechanism in the development of NeuroAids.

## Intercellular communication to brain ECs

### Activated immune cells release EVs that activate ECs

Since their discovery, EVs have been envisioned as a means of communication by which one cell type can affect specific cell types in other organ systems [57]. Much of how donor cell EVs communicate with recipient cells comes from in vitro studies in which the isolated EV subtype is added to the target cell. In general, EVs can affect inter-cellular communication by presenting ligands on their surface to receptors on the target cell plasma membrane. Alternatively, internalization of EVs can occur via either direct fusion of the EV membrane with the plasma membrane of the target cell, by phagocytosis and micropinocytosis, or by clathrin-mediated endocytosis. Endothelial cells, in particular, appear to use endocytic pathways rather than fusion mechanisms to uptake exosomes from different cell types [103]. In the study by Svenson et al. barrier forming HUVECs were shown to internalize exosomes from HeLa, U87MG, CHO and MEF cells by an endocytic process that followed a time, concentration, and temperature-dependent manner [103]. Although it is clear EVs can induce a wide variety of biological responses, little is known about the molecular mechanisms that transduce EV binding/uptake into actions by the recipient cell.

In the context of the brain endothelium, recent studies point to EV-mediated intercellular communication between immune cells and the cerebral vasculature. For example, experiments revealed EVs from activated monocytes stimulated human BMEC to upregulate adhesion molecules ICAM1 and VCAM1, the chemoattractant CCL2/MCP1 and pro-inflammatory cytokines IL6 and IL1β [104]. The authors further showed monocyte derived EVs may transfer miRNAs that can modulate endothelial activation. And though it has yet to be demonstrated whether transfer of miRNA by these exosomes can also regulate BBB status, work by Chugh et al. revealed EVs could promote endothelial migration [105]. Specifically, EVs from patients positive for Kaposi’s sarcoma enhanced the cellular migration of an immortalized cell line derived from HUVECs, suggesting a possible role for EVs in promoting angiogenic responses [105].

### Glioblastoma EVs induce BBB permeability

Glioblastoma is an extremely aggressive, highly vascularized brain tumor that is characterized by very poor prognosis and is associated with a large amount edema due to tumor-induced, vascular leakage [106]. This vascular leakage, at least in part, derives from the pro-permeability factor Semaphorin3A (Sema3A) produced within the glioblastoma, which triggers endothelial barrier permeability most commonly via engaging its receptor Neuropilin 1 (NRP1) [107]. Recently, it was shown that EVs released by patient-derived glioblastoma cells, and carrying Sem3A on their surface, can disrupt brain endothelial barrier function in vivo and in vitro in a NRP1-dependent manner [108], thus highlighting the role of EVs in mediating communication between the BBB and parenchymal cells.

### Platelet EVs/MPs to BMECs

Platelet EVs of the microvesicle/microparticle subtype have been reported to be internalized by cultured BMEC, entering both early endosome 1 antigen + endosomes and LysoTracker-labeled lysosomes, and altering the endothelial cell surface phenotype [109]. It has further been suggested such interaction(s) may play a crucial role in the pathogenesis of cerebral malaria by promoting the cytoadhesion of erythrocytes infected with the parasite *Plasmodium falciparum* to the brain endothelium [109, 110].

## Brain EC-derived EVs as serological biomarkers for BBB status during neuroinflammation

### EC-specific markers (CD31, Endoglin) likely on circulating EVs

#### Cerebral malaria

A key feature of cerebral malaria is the sequestration of *Plasmodium falciparum*-parasitized red blood cells (PRBC) within the brain microvasculature, leading to disturbances in microcirculatory flow, neuroinflammation and metabolic dysfunction [111, 112]. Consistent with inflammation and endothelial distress being signals for release of EVs from endothelial cells, EMP levels in plasma were observed to rise in Malawain children with severe malaria complicated with coma [113]. The pathogenicity of EVs was further demonstrated in a mouse model of cerebral malaria [114]. Inhibition of vesiculation and EV production conferred protection against neurologic disease, and adoptive transfer of fluorescently-labeled microparticles/microvesicles obtained from plasma of mice with CM resulted in these EVs lining the endothelium of brain microvessels of infected, but not healthy, recipient mice.

#### Ischemic stroke

Reflecting distress, EVs are elevated in vascular diseases in general, and in cerebrovascular accidents, like stroke, in particular [115] (for a concise review on the subject see Deng et al. [116]. Analysis of patients who suffered either a mild or moderate-severe stroke revealed higher levels of endothelial derived EVs when compared to non-stroke controls. This same study further reported the level of endothelial EVs correlated significantly with brain lesion volume, as measured by diffusion-weighted magnetic resonance imaging. In particular, EVs characterized by flow cytometry as CD105^+^CD54^+^CD45^−^ appeared to show the strongest correlation with lesion volume. Another study provided further support that endothelial EVs could effectively be used as biomarkers in the diagnosis for ischemic injury severity [117]. In this latter case, EVs isolated from 68 patients with acute ischemic stroke were compared to those isolated from age-matched controls. The results showed the elevated presence of CD31^+^ CD41a^−^ EVs in patients with acute ischemic stroke. Significantly, the profile of circulating endothelial EVs may offer a way to monitor and/or risk stratify cerebrovascular disease leading to stroke [115]. For example, individuals with intracranial arterial stenosis had higher levels of both CD31^+^ and CD42b^−^ EVs and CD31^+^ and annexin V^+^ microvesicles. No studies to date, however, have investigated whether any of these those populations of endothelial EVs contain proteins enriched at the brain endothelium. Such EVs may serve as useful surrogates for assessing the status of BBB in cerebrovascular disease and stroke.

#### MS

In light of release of endothelial EVs being associated with inflammation and endothelial distress, there has been considerable speculation as to whether they can serve as accessible biomarkers for MS—essentially “liquid biopsies” with potential diagnostic and prognostic value—as well as possible response indicators to treatment [7, 78, 118]. EMPs (identified by flow cytometry as < 3.0 um in diameter, and CD31^+^/CD42b^−^ or CD62E^+^) in platelet poor plasma (PPP) from patients with different clinical forms of MS—RRMS, primary progressive MS, secondary progressive MS, and CSI—were all found to be elevated when compared to healthy controls [119]. Significantly, MPs prepared in batch from PPP of RRMS patients, when compared to controls, significantly disturbed the integrity and barrier properties of cultured human endothelial cells (as reflected by diminished transendothelial electrical resistance) and altered these cells’ immunofluorescence staining of ZO-1 and the integral adherence junctional protein, VE-cadherin. Other reports have described changes in miRNA profiles of EVs—specifically exosomes—obtained from blood sera of MS patients [7, 120], but did not determine whether the EVs in question, in whole or in part, originated from endothelial cells.

#### Alzheimer’s disease

Cerebrovascular dysfunction is a hallmark of Alzheimer’s disease (AD) pathology. It thus stands to reason that in AD, endothelial remodeling and inflammatory responses would yield endothelial derived EVs. In fact, levels and marker profiles of endothelial EVs in the plasma of AD patients have been found to differ from those of healthy controls [121]. In the study by Xue et al. significant increases in the levels of CD31^+^CD42^−^ and CD62e^+^CD42^−^ EVs, respectively, were detected in the AD group relative to the controls. However, the elevation in EVs did not showed a difference as a function of AD severity (mild to severe) groups. In another study, a comparative endothelial EV analysis was performed in AD patients with and without vascular risk factors, such as hypertension, diabetes, dyslipidemia, stroke, coronary artery disease and smoking. Interestingly, this study did not indicate a difference in EV levels between AD patients and controls. However, a significant difference did appear between AD patients diagnosed with vascular risk factors and those without [122]. This topic clearly warrants further study, in which EV phenotyping could be expanded to include other surface markers, monitored in different forms of dementias and assessed in longitudinal studies.

#### Traumatic brain injury (TBI)

TBI represents a major public health concern with long lasting effects on both the patient and surrounding communities (i.e., long-term care). Currently, there are no clinically approved biomarkers to diagnose TBI or monitor the recovery [123]. However, several neuronal or astrocytic markers have been examined including S100β, neuron-specific enolase, glial fibrillary acidic protein, and Tau [124]. Consequently, the use of brain endothelial biomarkers represents a largely unexplored avenue for biomarker discovery. It is well established that TBI causes cerebral vascular damage both during the primary phase (induced by forces that are linear or rotational) and the secondary phase (a result of inflammation). These resulting changes in vascular integrity and BBB permeability, in some cases, can last for months or years following the original injury [125] and can result in delayed neuronal death and dysfunction [126]. This timeline for endothelial damage may suggest the use of brain EC-derived vesicle biomarkers over the course of both phases of TBI pathology. Some initial studies have examined the detection of endothelial-enriched markers following TBI. First, elevated levels of the TJ protein occludin were detected in patient serum following a mild TBI [127]. Around that same time, Andrews et al. sought to demonstrate that brain ECs release EVs in response to mechanical injury and that these vesicles contain TJ proteins such as occludin. Using an in vitro model, commonly used to simulate TBI on neurons, they showed that BMECs released vesicles containing PECAM-1, ICAM-1 and occludin. They further confirmed the elevated release of vesicles containing occludin in serum following TBI in vivo by flow cytometry and cryo-EM [45]. However, in both these studies, the authors did not delineate EVs released from the endothelium versus the epithelium—both of which express occludin. However, they do offer proof of principal for the detection of brain EC-enriched EV proteins as possible biomarkers. Future studies using dual or a combination of markers would be able to identify BBB specific vesicles and the potential for monitoring the recovery of the vasculature over time following TBI.

## Role of EVs derived from other CNS barriers

### Blood–spinal cord barrier (BSCB)

Largely analogous to the BBB in function and structure, with regard to restricting the passage of soluble and cellular elements between the blood circulation and CNS parenchyma, the BSCB nevertheless exhibits some distinguishing features compared to its brain counterpart [128, 129]. These include greater inherent permeability [130] and heightened susceptibility to stimuli that regulate endothelial integrity [131]. Such differences might at least partially stem from comparatively reduced expression of TJ proteins occludin and ZO-1 by spinal cord microvascular endothelial cells [27]. EV interactions with the spinal cord microvasculature should thus be viewed as possibly reflecting unique spinal endothelial properties. In this respect, pharmacologic inhibition of exosome production by pericytes, which lie adventitial to the endothelium and are integral to the NVU of both the BBB and BSCB, was found to preclude these cells’ ability to promote angiogenesis when placed in co-culture with a spinal cord explant [132]. Insofar as cross-talk via soluble mediators between pericytes and CNS microvascular endothelial cells is critical for maintenance of barrier properties and regulation of inflammation [133, 134], pericyte-derived EVs might also be fundamental to these processes.

### Blood–cerebrospinal fluid barrier (BCSFB)

The CSF space, occupied by the brain ventricles, subarachnoid spaces (SAS) of the brain and spinal cord, and central spinal canal, is separated from the blood circulation by the BCSFB. At the level of the choroid plexus (CP), which projects into the brain ventricular system and is a major producer of CSF, the BSCFB lies at the choroidal epithelium—a high resistance layer of simple columnar epithelial cells possessing tight junctions and a series of asymmetrically positioned ion transporters, enzymes and receptors that facilitate production of CSF. This epithelium encapsulates a vascular stroma comprised of fenestrated capillaries lacking typical BBB properties [135]—an arrangement establishing the BCSFB. The lateral foramina of Lushka and medial foramen of Magendie of the brain allow direct communication between the CSF in ventricles and that in the SAS. Evidence has accumulated that choroidal epithelial cells exploit EVs to shuttle specific cargo through the CSF to distant CNS regions. An example of this is the delivery of folate to the brain [136]. The folate receptor (FRα) on that basolateral surface of choroidal epithelial cells (i.e., the side facing the stromal capillaries) can pick up extravasated folate and, following endocytosis, be incorporated (complexed with folate) into MVBs. MVBs containing the FRα-folate complex can then fuse with the apical membrane of the choroidal epithelial cells, thereby releasing the FRα-folate complex as folate-bearing exosomes into the CSF. Once in the CSF, these exosomes can be delivered across the ependymal cells lining the brain ventricles and into the parenchyma. Additionally, EVs released by CP choroidal epithelial cells appear to play a role in relaying inflammatory signals from the periphery to the CNS. When peripherally injected into mice, LPS, a component of the cell wall of Gram negative bacteria, typically induces florid inflammation similar to that seen in sepsis [137]. However, it was recently shown that systemic LPS injection resulted in a large increase in nascent exosomes (in the form of intraluminal vesicles) within MVBs in CP choroidal epithelial cells [138]. Contemporaneous elevation of exosomes bearing by the CP marker, transthyretin, was detected in the CSF, suggesting the CP is an important source of EVs during system inflammation. This rise in CSF exosomes further correlated with changes in expression in both CSF and CP of miRNAs associated with inflammation: miR-1a and miR-9 were significantly down-regulated, while miR-146a and miR-155 were significantly up-regulated. And, intracerebroventricular injection of GW4869, an exosome inhibitor, reduced the amount of EVs in the CSF while causing accumulation of miRNA-146a, miRNA-155, and miR-9 in the CP. These LPS-elicited EVs were additionally observed to cross the ependymal cells and penetrate into the brain parenchyma, where they located in close proximity to GFAP + astrocytes. Studies with primary mixed cortical cultures confirmed this EV: astrocyte affinity and further identified GFAP + astrocytes and IBA1 + microglia as target cells that can take up these EVs and, as a consequence, suffer downregulation of a number of genes linked to inflammatory pathways. This last series of findings offer strong support for the following scenario: EVs are produced by the BCSFB and deposited into the CSF in response to peripheral challenges, travel via the CSF to target sites, and then transmit their miRNA cargo into recipient parenchymal neural cells to effect gene regulation within the CNS [138].

### Blood–retinal barrier (BRB)

The retinal pigmented epithelium (RPE) forms the outer BRB between the systemic circulation (on its basolateral side) and the retina (on its apical side) [139]. Cultured RPE cells grown in dual-chamber Transwell format were shown to release exosomes in a polarized manner; of 631 proteins identified by protein correlation profiling mass spectrometry, 299 were uniquely released apically, while 94 uniquely released basolaterally. It has further been posited that exosomes secreted by RPE cells serve as a mode of communication between the RPE and outer retina [140]. In a model system using posterior poles with retina removed from fresh human donor eyes, L-DOPA stimulation of GPR143, a G-protein couple expressed by RPE cells that contributes to pigmentation of eyes and skin, inhibited constitutive release of RPE exosomes. Concomitantly, myocilin, a protein involved in regulating intra-ocular pressure and that interacts with GPR143 in a signal transduction-dependent manner, was recruited to the endocytic compartment, indicating GPR143 and myocillin cooperate in a signal transduction pathway that regulates RPE exosome release. EVs can also act pathologically at the BRB, as EVs from mesenchymal stem cells that were cultured under diabetic-like conditions were shown to elicit features of diabetic retinopathy by elevating miR-126 expression in pericytes and causing up-regulation of angiogenic molecules VEGF and HIF-1a [141].

## Use of EVs for therapeutic applications

### Mediators to improve vascular pathology

The promise of EVs for therapeutic applications has sparked much interest in recent years. Much of the work thus far has focused on evaluating the therapeutic use of EVs collected from mesenchymal stem cells (MSCs) or human induced pluripotent cell–derived mesenchymal stem cells (hiPSC–MSCs) [142] (see Samsonraj et al. [143] for a recent review). EVs used as therapeutic agents have, in large, been tested in the context of tissue regeneration and wound healing. In terms of vascular injury, it is noteworthy that EVs from nearly every cell type appear to affect angiogenic responses; this includes EVs derived from cardiac progenitor cells, adipose-derived stem cells, and peripheral blood mononuclear cells to name a few. Although the positive effects of EVs on the endothelium remains unknown, it is thought that revascularization and survival factors produced by triggering the angiogenic program is a key component for recovery and tissue regeneration.

MSCs are generally recognized as adult stem cells that are able to give rise to mature mesenchymal cell types, e.g., osteoblasts, adipocytes and chondrocytes, and secrete a wide variety of bioactive molecules that display properties with considerable potential therapeutic importance: anti-apoptotic, anti-inflammatory, anti-scarring, angiogenic and chemotactic [144]. As such, there is great tendency to exploit them for their reparative capabilities in conditions where CNS barriers are disturbed [143].

MSC-derived EVs have been shown to mediate a variety of therapeutic effects [145–147], revealing their strong immunomodulatory role(s). Suggestive reparative actions of EVs at CNS barriers have also emerged as shown by examples of EV-driven remediation of vascular-associated brain injury [148, 149]. For example, multipotent human bone marrow-derived MSCs improved functional recovery after traumatic brain injury in rats, in part, by stimulating angiogenesis and neurogenesis, while also suppressing neuroinflammation [150]. In a preclinical model of hypoxic-ischemic brain injury in ovine fetuses, in utero intravenous administration of MSC-EVs reduced the total number and duration of seizures and preserved baroreceptor reflex sensitivity [151]. And, two-repetitive doses of MSV-derived EVs were found to attenuate effects of intraperitoneal injection of LPS, significantly lessening inflammation-induced neuronal cellular degeneration and microgliosis, restoring myelination and reversing long-term microstructural abnormalities of the white matter, and improving lost-lasting cognitive functions [152].

Perhaps the ability of MSC-derived EVs to execute therapeutic effects on the brain and CNS barriers stem from the capacity of EVs to intimately interact with and cross the BBB. Transwell assays employing BMECs, revealed luciferase-carrying exosomes from HEK293 cells were internalized by BMECs via endocytosis, co-localized with endosomes, and exploited the transcytotic pathway [153]. Significantly, BMEC transit only occurred when BMEC were placed under a stroke-like, inflamed condition by pre-treatment with TNF-α, suggesting EVs may remediate brain injury best under situations where the BBB is already compromised [153].

To date, there have been but a few reports on the therapeutic potential of EVs derived from BMECs. Kurachi et al. demonstrated the advantageous outcome that these EVs have on oligodendrocyte precursor cells (OPC) [63]. In this study, the investigators observed EVs derived from rat BMEC promoted proliferation and motility of cultured rat OPCs. The group additionally determined that endothelial EVs from other vascular beds or from human also shared the potential to promote OPC survival. As the authors postulated, perhaps it is via release of EVs that transplanted BMECs confer protection to OPCs in an animal model of ischemic white matter infarct [154]. These pioneering studies thus offer promise of endothelial EV based therapies for the treatment of demyelinating diseases. It is also intriguing to consider BMEC-generated EVs may represent an untapped therapeutic resource for neuroprotection, anti-inflammation and regeneration.

### Drug carriers/delivery agents

A major obstacle in the treatment of CNS diseases is the high rate of failure of systemically administered drugs to effectively cross the BBB and reach therapeutic levels in the brain parenchyma [155]. Thus, there is a dire need to discover new, or modify existing, delivery vehicles that can successful encapsulate a therapeutic agent and deliver it to the CNS. Recent developments in CNS drug delivery have taken advantage of the fundamental properties of EVs to package cellular proteins and genetic material from the host cell. In this way, EVs add to the toolbox of nanomedicine, viral constructs and liposome platforms [156]. EVs make excellent delivery vehicles for various reasons. First, as reported, immunogenicity of EVs is extremely low, which helps increase bioavailability [157]. Second, cellular targeting can be controlled since ligand composition of EV membranes can be engineered or selected based on the donor cell of origin. Experiments reported by Liu et al. provide an example of how EVs are used in this manner. Employing EVs generated from human neural progenitor cells (NPC), Liu and colleagues demonstrated the addition of NPC EVs loaded with miR-210 protected human brain endothelial cells from insult [158]. Specifically, miR-210, a known mediator of antioxidant endothelial responses, when internalized by BMECs, provided endothelial protection against angiotensin II-induced ROS overproduction and EC dysfunction. In addition to mRNA, miRNA and siRNA loading of EVs, recombinant protein delivery to the CNS via EVs has also shown promising results. For example, in a model for the neurodegenerative disorder cerebral folate transport deficiency, investigators showed folate receptor-α containing EVs injected via the intraventricular route restored folate transport [136]. The therapeutic potential of including recombinant proteins in EV cargo was also displayed in a report in which recombinant catalase was captured in macrophage derived EVs and administered intranasally or intra-venously in a Parkinson’s mouse model [159]. Injection of these EVs provided considerable protection to dopaminergic neurons. Drug loading of EVs, a procedure often used in the context of anti-cancer treatment, has been demonstrated using many different types of cells [160]. Also, a strategy combining liposome-containing hydrophobic compounds in EVs has been devised as a means to potentially package anti-tumor compounds [161]. The latest novel utilization of EVs as delivery vehicles includes the encapsulation of AAV gene therapy vectors (vexosomes) for the treatment of CNS diseases [162, 163]. These vexosomes not only can target the CNS, but also enable stable transgene expression by the AAV component [156–160].

Although many challenges still remain, such as loading efficiency, specific targeting, scalability and batch to batch consistency, promising results so far are clearly paving the way for the utility of EVs as CNS delivery vehicles.

## Perspectives and future directions

### As biomarkers

The potential for using EVs as biomarkers in diseases remains of great interest. However, key questions remain regarding how EV subtype and composition change as a function of disease or recovery. Furthermore, can a “signature” EV (i.e., panel in a vesicle biomarker) be identified with the potential to specifically identify a disease or disease severity especially between two conditions with similar pathology? Observations in a recent study found that while 4 markers were increased in both mild TBI and orthopedic patients (compared to healthy controls) only the combination of galectin-3 and occludin was able to distinguish between the two injury types [164]. It is important to note in this study that the authors did not isolate EVs; hence, these markers may be soluble or EV-associated. In some cases, it may be unnecessary to have a biomarker that can distinguish between two related diseases such as TBI and stroke, which have very defined initiation events. However, co-occurring events (i.e. TBI and orthopedic injury) may require more unique biomarkers to identify the prognosis of each injury. In addition, slowly progressing diseases may be harder to discern against the background of individual baselines or noise from multiple conditions. Many initial studies into biomarker utility are restricted and low in power, and the noted effects may become negligible when investigations are expanded. Overall, a panel approach of various biomarkers, along with large-scale multicenter longitudinal approaches will be needed to fully evaluate the utility of blood biomarkers in diagnosing and monitoring recovery following disease or injury onset [124].

### In pathology

(i) Do different immune cell subtypes favor brain EVs with a particular TJ protein composition for CNS entry? And, is the act of TJ protein^+^ EV: leukocyte binding determined by activation state of the leukocyte, or does it determine leukocyte activation state? These questions remain to be answered, and results will shed necessary light on the role(s) of these EVs during pathology. Observations that both circulating EVs and leukocytes display TJ proteins in neuroinflammatory conditions [44, 45, 95], and leukocytes receive at least some of their TJ protein from endothelial cells possibly through EV transmission [44], have prompted suggestion EV:leukocyte communication can regulate leukocyte extravasation into the CNS through a TJ protein-mediated zipper mechanism. However, it remains to be determined that such a scenario operates. As leukocyte TEM occurs both through intercellular (between cells) and transcellular (through cells) routes, EV interactions with particular leukocyte subtypes might influence which route prevails. In this regard, it’s been hypothesized that different leukocyte subtypes prefer one route over the other [165], and the extent of leukocyte activation may yet be another determining factor [166]. Future studies detailing whether specific leukocyte subtypes bind EVs containing specific TJ or other adhesion proteins, how this binding may be related to leukocyte activation state, and what effect this binding has on leukocyte TEM, could provide insight into which route of TEM is preferred—and why.
(ii) Do EV interactions at the BBB regulate tumor metastasis to the brain? Given EVs released from varied cell types can impact BMEC gene expression, adhesive properties, and permeability in vitro and in vivo [108], it is reasonable to consider EVs derived from tumor cells could potentially manipulate the BBB to support brain metastasis. Indeed, EVs (comprised of exosomes and microvesicles) isolated from culture supernatants of human breast cancer cells having brain high-metastatic potential were shown to trigger the breakdown of the BBB though a miR-181c-mediated action that interferes with subcortical action dynamics in BMEC (demonstrated using a monkey-derived culture model), promote brain metastasis of breast cancer cell lines, and become preferentially incorporated into the brain of mice in vivo [167]. And it has further been demonstrated that exosomes from mouse and human brain tropic tumor cells, when injected into mice, fuse preferentially with brain endothelial cells [168]. These findings highlight the prospect that interfering with EV release may have therapeutic potential in cancer treatment by mitigating brain involvement.
(iii) Does inhibition of EV biogenesis confer BBB protection? To effectively pursue this question, it is important to recognize acknowledge that certain steps in EV biogenesis are universal for all cell types, while others are not [169]. Thus, answering how EV biogenesis in a brain ECs differ from other cell types or from ECs in other vasculature are likely to produce key insight into how this important biological process can be regulated in diseases of the CNS. Perhaps as a starting point, known pharmacological agents that appear to inhibit EV production could be tested. To date, no reports can be found in which inhibition of EV release has been tested as a therapeutic avenue in the context of neuroinflammation. Should EVs facilitate TEM, it stands to reason that interference with EV release would mitigate neuroinflammation and sustain BBB integrity. However, in support for the idea that interference of EV biogenesis can provide beneficial outcomes, is the utilization of GW4869 in experimental sepsis [170]. Specifically, in vivo inhibition of exosome biogenesis/release by injection of GW4869, was found to lower amounts of exosomes and pro-inflammatory cytokines in serum, and diminish sepsis-induced inflammation and myocardial depression following systemic LPS treatment or cecal ligation/punction surgery in mice [170]. GW4869 administration also alleviated various asthmatic features in a rodent disease model [171], and blocked exosomes from mature dendritic cells from contributing to endothelial inflammation [172], additionally arguing that suppression of EV biogenesis/release attenuates inflammatory-associated disruption to epithelial/endothelial tissues. This raises the prospect that interfering with EV:leukocyte interaction—perhaps by targeting TJ proteins shared by both these elements and BBB endothelial cells—could block formation of EV:leukocyte complexes that have enhanced TEM capability [36, 77] and, thereby, frustrate leukocyte invasion of the CNS.

## Conclusions

Just as the complexity of the endothelium—once conventionally dismissed as functionally inert—has become widely recognized, so too has appreciation of the physiological and pathophysiological importance of EVs burgeoned rapidly. What might have begun as “dust” is unlikely to return to dust any time soon. With increased understanding of their association with neuroinflammatory and neurodegenerative disease, EVs are providing a window on the world of CNS barrier status in these conditions. Accessibility of biological fluids is key, as acquisition of EVs from these compartments is minimally invasive and can be performed repeatedly. While blood has most commonly been analyzed, and is a depot of EVs released from all circulating blood elements as well as the luminal surface of the endothelium, EVs present in the CSF are also considered “biomarker treasure chests” [173]. In particular, CSF EVs are likely to more accurately reflect activity of the choroidal epithelial cells of the BCSFB and leptomeningeal cells of the BLMB, as well as immune cell enterprises within the meninges [174]. Improvements in isolating EVs from small amounts of biological fluids and CNS tissues [175], and resolving EV heterogeneity by using high sensitivity/high resolution flow cytometry and cell surface markers to identify donor cell types [176, 177], will prove crucial to realizing the full impact of EVs in the physiology and pathophysiology of CNS barriers.

## Abbreviations

AAV: adeno-associated virus
AD: Alzheimer’s disease
ARF6: ADP-ribosylation factor 6
ART: antiretroviral therapy
BBB: blood–brain barrier
BCSFB: blood–cerebral-spinal-fluid barrier
BLMB: blood–leptomeningeal barrier
BMEC: brain microvascular endothelial cell
BRB: blood–retinal barrier
CLN-5: claudin-5
CM: cerebral malaria
CP: choroid plexus
CNS: central nervous system
CSI: clinically isolated syndrome
EAE: experimental autoimmune encephalomyelitis
EC: endothelial cell
EMPs: endothelial microparticles
EV: extracellular vesicles
FRα: folate receptor
HAND: HIV-1-associated neurocognitive disorders
hiPSC-MSCs: human induced pluripotent cell-derived mesenchymal stem cells
MP: microparticle
MS: multiple sclerosis
MRI: magnetic resonance imaging
MSC: mesenchymal stem cell
MV: microvesicle
MVB: multi-vesicular bodies
NPC: neural progenitor cells
NRP1: Neuropilin 1
NVU: neurovascular unit
OPC: oligodendrocyte precursor cells
Pgp: p-glycoprotein
PPP: platelet poor plasma
PRBC: parasitized red blood cells
ROS: reactive oxygen species
RPE: retinal pigmented epithelium
RRMS: relapsing/remitting MS
SAS: subarachnoid space
TEM: transendothelial migration
TJ: tight junctions
TBI: traumatic brain injury
vWF: von Willibrand factor
